# Amyloid precursor protein facilitates SARS-CoV-2 virus entry into cells and enhances amyloid-β-associated pathology in APP/PS1 mouse model of Alzheimer’s disease

**DOI:** 10.1038/s41398-023-02692-z

**Published:** 2023-12-16

**Authors:** Jiang Chen, Junsheng Chen, Zhifeng Lei, Fengning Zhang, Ling-Hui Zeng, Ximei Wu, Song Li, Jun Tan

**Affiliations:** 1 Department of Pharmacology, Zhejiang University School of Medicine, 310058 Hangzhou, China; 2 Key Laboratory of Novel Targets and Drug Study for Neural Repair of Zhejiang Province, School of Medicine, Hangzhou City University, 310015 Hangzhou, Zhejiang China; 3 Key Laboratory of Endemic and Ethnic Diseases, Laboratory of Molecular Biology, Ministry of Education, Guizhou Medical University, 550025 Guiyang, Guizhou China; 4 First Affiliated Hospital of Dalian Medical University, 116021 Dalian, Liaoning China

**Keywords:** Molecular neuroscience, Pathogenesis

## Abstract

Although there are indications of a trend towards less severe acute respiratory symptoms and a decline in overall lethality from the novel Coronavirus Disease 2019 (COVID-19) caused by Severe Acute Respiratory Syndrome Coronavirus 2 (SARS-CoV-2), more and more attention has been paid to the long COVID, including the increased risk of Alzheimer’s disease (AD) in COVID-19 patients. In this study, we aim to investigate the involvement of N-terminal amyloid precursor protein (APP) in SARS-CoV-2-induced amyloid-β (Aβ) pathology. Utilizing both in vitro and in vivo methodologies, we first investigated the interaction between the spike protein of SARS-CoV-2 and N-terminal APP via LSPR and CoIP assays. The in vitro impacts of APP overexpression on virus infection were further evaluated in HEK293T/ACE2 cells, SH-SY5Y cells, and Vero cells. We also analyzed the pseudovirus infection in vivo in a mouse model overexpressing human wild-type APP. Finally, we evaluated the impact of APP on pseudovirus infection within human brain organoids and assessed the chronic effects of pseudovirus infection on Aβ levels. We reported here for the first time that APP, the precursor of the Aβ of AD, interacts with the Spike protein of SARS-CoV-2. Moreover, both in vivo and in vitro data further indicated that APP promotes the cellular entry of the virus, and exacerbates Aβ-associated pathology in the APP/PS1 mouse model of AD, which can be ameliorated by N-terminal APP blockage. Our findings provide experimental evidence to interpret APP-related mechanisms underlying AD-like neuropathology in COVID-19 patients and may pave the way to help inform risk management and therapeutic strategies against diseases accordingly.

## Introduction

Since the outbreak of the novel Coronavirus Disease 2019 (COVID-19) caused by Severe Acute Respiratory Syndrome Coronavirus 2 (SARS-CoV-2), ~6.8 million people have died and the total number of global confirmed cases was over 750 million as of 1 February 2023 (https://covid19.who.int/). SARS-CoV-2 tropism and entries are predominantly mediated by the interaction between coronavirus spike (S) [1] protein and corresponding host receptors, especially the angiotensin-converting enzyme 2 (ACE2) [2–6]. Binding of SARS-CoV-2 to ACE2 occurs via the receptor-binding domain of the S1 subunit of the S protein [5, 7, 8]. Interestingly, HDL-scavenger receptor B type 1, tyrosine-protein kinase receptor UFO, neuropilin-1, a disintegrin and metalloproteinase (ADAM), and apolipoprotein E have been reported to facilitate SARS-COV-2 infection [9–13]. In addition, asialoglycoprotein receptor 1 and kringle-containing transmembrane protein 1 have recently been reported to serve as SARS-CoV-2 receptors to impact viral infection and antibody-mediated neutralization [14]. Moreover, SARS-CoV-2 infects T-lymphocytes, platelets, and megakaryocytes via ACE2-independent mechanisms [15, 16]. All these findings suggest that ACE2 is not working solely during SARS-CoV-2 infection and the host cells entry of SARS-CoV-2 is a multi-receptor process.

While the incidence of serious acute respiratory symptoms related to this global pandemic is declining gradually, and unexpectedly, emerging clinical reports indicate that neurological manifestations continue to rise, suggesting detrimental impacts of SARS-CoV-2 on the central nervous system (CNS) [17–23]. Notably, reports about cerebral involvement among patients infected, as well as identification of viral infection in the brain suggested that these coronaviruses may directly infect the CNS. Increasing lines of evidence have implicated the possible impacts of SARS-CoV-2 infection on AD-like pathologies [22, 24–26], including the increased amyloid precursor protein (APP) expression [27, 28], exacerbated β-amyloid (Aβ) pathology [29], and elevated serum level of phosphorylated tau [30]. Moreover, imaging neurons of organoids reveals that SARS-CoV-2 exposure is associated with altered tau hyperphosphorylation and apparent neuronal death [31]. Collectively, these findings hint at the long-term neurodegenerative effects of SARS-CoV-2. Given that cell entry of SARS-CoV-2 may involve multiple transmembrane receptors and the close correlations between COVID-19 and AD, we speculate that AD-related proteins may mediate the cerebral infection of SARS-CoV-2.

APP, as the key regulatory hub of AD [32, 33], is a single-pass transmembrane protein abundantly expressed in the brain and metabolized in a rapid and highly complex fashion by a series of sequential proteases, including the ADAM10, ADAM17, β-site APP cleaving enzyme-1 and γ-secretase complex, which also process other key regulatory molecules [34]. Genetic, biochemical, and behavioral studies have documented that physiologic generation of the neurotoxic Aβ peptide from sequential APP proteolysis is the crucial step in the development of AD [35]. Moreover, the large ectodomain of APP, which has recently been revealed to be multifunctional, plays receptor-like roles. For instance, Liu et al. found that a conserved cysteine-rich domain in the extracellular portion of APP protein is required for Wnt binding, which promotes APP recycling and stability [36]. ApoE has also been found to interact with N-terminal APP to exacerbate AD pathologies, which can be reversed by 6KApoEp, an N-terminal APP antagonist peptide [37]. Worth noting, herpes simplex virus (HSV) has been reported to interact with APP to promote its transport [38]. Much more interestingly, HSV type-1 binding to the heparan sulfate proteoamino glycans expressed on neuronal plasma membrane induces Ca^2+^ signals triggering APP phosphorylation at Thr668 that, in turn, increases β- and γ-secretase activity [39]. As a result, there is an increased production of Aβ that aggregates to form oligomers of various sizes [40]. In addition, the human immunodeficiency virus (HIV-1) polyprotein Gag has been reported to interact with APP in membrane lipid rafts [41]. Overall, APP has emerged as a critical receptor or co-factor that affects virus entry; however, the potential role of APP in SARS-CoV-2 infection is still unknown. Here, we investigated whether APP is a binding target of SARS-CoV-2 that mediates its host cell infection. We found that APP interacts with the S protein of SARS-CoV-2, promotes the cellular entry of the virus, and exacerbates Aβ-associated pathology in the APP/PS1 mouse model of AD, which can be inhibited by N-terminal APP blocker AY51, a peptide analog of 6KApoEp [32, 33]. These findings provide direct experimental evidence to interpret APP-related mechanisms underlying AD-like neuropathology in COVID-19 patients and may pave the way to help risk management and discover therapeutic strategies accordingly.

## Results

### SARS-CoV-2 S protein highly binds to N-terminal APP

**Fig. 1:**
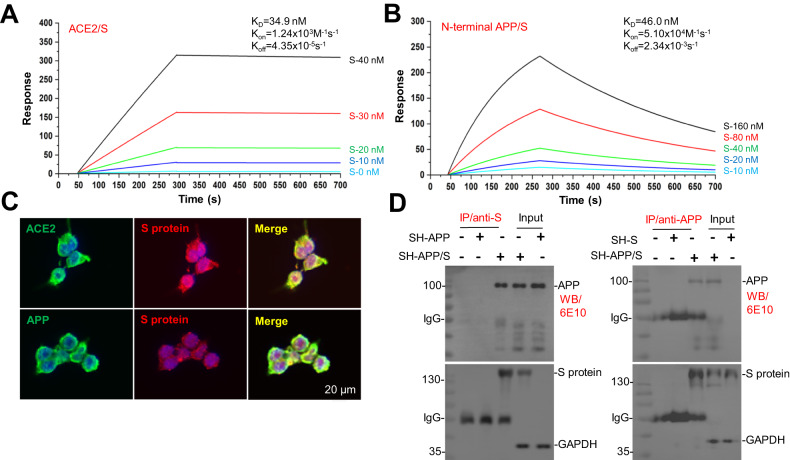
SARS-CoV-2 spike (S) protein highly binds to N-terminal amyloid precursor protein (APP). **A** and **B** ACE2 protein or N-terminal APP (18-612 amino acids, APP18-612), captured on the COOH chip, can bind to S protein with an affinity constant of 34.9 or 46.0 nM, respectively, as determined by localized surface plasmon resonance (LSPR) assay. **C** Human embryonic kidney 293T cells (HEK293T) stably overexpressing either hACE2 (HEK293T/ACE2) or hAPP (HEK293T/APP) were plated in confocal chambers at a density of 1 × 10^5^/well for 24 h. The cells were transfected with constructs encoding SARS-CoV-2 S protein for 48 h, then fixed in 4% paraformaldehyde solution and subjected to immunofluorescence staining with anti-hACE2 antibody, anti-hAPP antibody (6E10), and anti-S protein antibody. Alexa Fluor 488 goat anti-mouse immunoglobulin G (IgG) was used to detect hACE2 (green) or hAPP (green), while Alexa Fluor 555 donkey anti-rabbit IgG was used to detect S protein (red). DAPI (4’,6-diamidino-2-phenylindole) is used as a nuclear counterstain (blue) and visualized by confocal microscopy. Scale bar 20 μm. **D** Validation of the interaction between SARS-CoV-2 S protein (136.9 kDa) and N-terminal APP protein. SH-SY5Y cells were co-transfected with SARS-CoV-2 S protein and APP constructs for 48 h. S protein antibody or APP antibody were primarily incubated with MAg25K protein A/G agarose beads (1 h at 37 °C) to prepare the agarose beads antibody complex. The agarose beads antibody complex was then incubated with whole-cell lysates overnight at 4 °C, followed by western blotting (WB) with anti-APP antibody (6E10); An immunoprecipitation (IP) experiment in the reverse direction followed by WB with anti-S protein antibody was performed to further confirm the association of N-terminal APP protein with S protein. IP with mouse serum was used as a blank control and the lysate as the input control.

To confirm the possible interaction between S protein and N-terminal APP, a localized surface plasmon resonance (LSPR) assay was employed. The binding affinity of S protein with ACE2 was also determined for reference. Various concentrations of S protein (10–160 nM) or ACE2 (0–40 nM) were injected into an LSPR sensor chip, on which recombinant APP N-terminal (18-612) (APP18-612) fragment was pre-coated. As shown in Fig. 1A, the LSPR assay confirmed the interaction of S protein with ACE2 with the equilibrium dissociation constant (KD) of 34.9 nM. Interestingly, our data also identified the interaction of S protein to APP18-612 in a concentration-dependent manner (Fig. 1B), with a KD of 46 nM. Worth noting, compared with ACE2, APP18-612 showed a relatively weaker binding affinity and a gradual dissociation from S protein, as evidenced by the lower KD value and a falling tendency of response curve after 275 s. APP specifically binds to ApoE4 with detailed kinetic parameters, whereas APP or Tau does not bind to BSA, indicating specificity (Supplementary Fig. 1A–C).

In addition, the interaction between APP and S was further evaluated in vitro by using immunofluorescence (IF) staining in Human embryonic kidney 293T cells stably overexpressing human WT APP695 (HEK293T/APP) or overexpressing human ACE2 (HEK293T/ACE2), both transiently transfected with S protein. As expected, clear co-localizations of cellular APP and S were presented (Fig. 1C, bottom images; Supplementary Fig. 1D, middle images), suggesting direct interactions between these two proteins. ACE2 and S as positive control also showed clear co-localizations (Fig. 1C and Supplementary Fig. 1D, upper images). CAV1 and S as negative control also showed clear co-localizations (Supplementary Fig. 1D, bottom images).

Moreover, the interactions between APP and S protein were further confirmed using immunoprecipitation (IP) analysis. Cell lysates prepared from human SH-SY5Y neuroblastoma cells stably overexpressing human WT APP695 (SH-SY5Y/APP695) or SH-SY5Y cells overexpressing both APP695 and S (SH-SY5Y/APP695/S) were immunoprecipitated using anti-S protein antibody, and then APP and S proteins were determined using western blot (WB) with 6E10 (anti-N-terminal APP; Fig. 1D, *left panel*) or S protein antibody (anti-S; Fig. 1D, *right panel*); Clear interactions between cellular APP and S protein can be observed.

### Overexpression of APP enhances SARS-CoV-2 pseudovirus infection in vitro

**Fig. 2:**
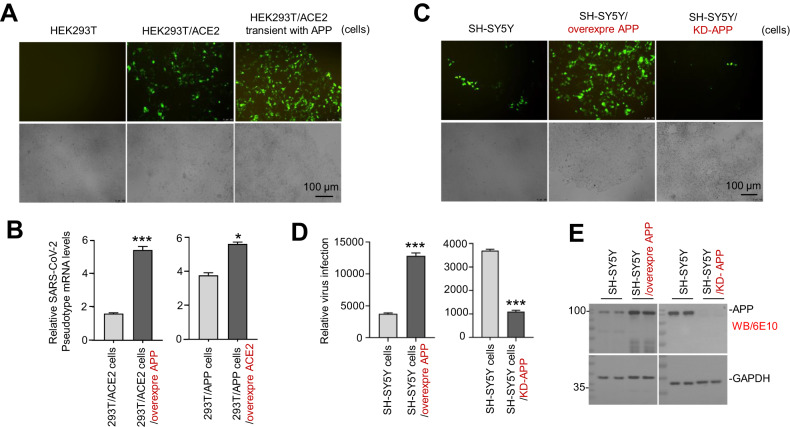
Overexpressing APP promotes SARS-CoV-2 pseudovirus infection in HEK293T and SH-SY5Y cells. **A** and **B** APP facilitates pseudovirus (Pseudo-SARS-CoV-2 virus) infection. **A** HEK293T, HEK293T/hACE2, and HEK293T/hACE2 transiently transfected with constructs encoding APP (HEK293T/hACE2 transient with APP) were plated in six-well chambers at a density of 1 × 10^5^/well for 24 h. Cells were infected with the GFP-labeled pseudovirus at 10^8^/mL for 48 h at 37 °C, and visualized by fluorescence microscopy. Scale bar 100 μm. **B** HEK293T/hACE2 cells transfected with constructs encoding hAPP or HEK293T/ hAPP cells transfected with constructs encoding hACE2 were infected with the GFP-labeled pseudovirus at 10^8^/mL for 72 h at 37 °C. Pseudovirus mRNA from cell lysis was examined by RT-qPCR. The data are shown as the means ± SEM from three independent experiments. *P* values were calculated using two-way ANOVA (*P < 0.05; ***P < 0.001). **C** SH-SY5Y cells, SH-SY5Y cells stably overexpressing hAPP and SH-SY5Y cells with APP knockdown were infected with the GFP-labeled pseudovirus at 10^8^/mL and visualized by fluorescence microscopy at 48 h post-infection. Scale bar 100 μm. **D** Pseudovirus from cells lysis in **C** was examined by RT-qPCR. The data are shown as the means ± SEM from three independent experiments. *P* values were calculated using two-way ANOVA (***P < 0.001). **E** The APP overexpression and knockdown efficiencies in SH-SY5Y were evaluated using WB assay with anti-APP antibody (6E10). GAPDH was selected as a reference control.

As expected, compared with HEK293T cells without endogenous ACE2 expression, HEK293T/ACE2 cells overexpressing exogenous ACE2 can be successfully infected by GFP-labeled pseudovirus, as evidenced by the increased GFP green fluorescence (Fig. 2A). In addition, the HEK293T/ACE2 cells with transient APP overexpression showed a further dramatic increase of green fluorescence, indicating a much higher sensitivity to pseudovirus infection than those HEK293T/ACE2 cells and a synergism between APP and ACE2. qRT-PCR assay further confirmed the elevated intracellular pseudovirus load in either HEK293T/ACE2 cells with APP overexpression (Fig. 2B) or HEK293T/APP cells with ACE2 overexpression (Fig. 2B), compared with the HEK293T/ACE2 (*P* < 0.001) or HEK293T/APP cells (*P* < 0.05), respectively.

Consistent with HEK293T/ACE2 cells, SH-SY5Y cells expressing endogenous ACE2 also showed an infectibility to pseudovirus, which can be strengthened by the overexpression of APP in SH-SY5Y/APP cells (*P* < 0.001; Fig. 2C, D). Interestingly, pseudovirus infection can be significantly blocked by endogenous APP knockdown (KD) in the SH-SY5Y/KD-APP cells (*P* < 0.001; Fig. 2C, D). The expression levels of APP in SH-SY5Y/APP and SH-SY5Y/KD-APP cells were confirmed by WB (Fig. 2E).

### In vivo expression of APP promotes SARS-CoV-2 pseudovirus infection in mice

**Fig. 3:**
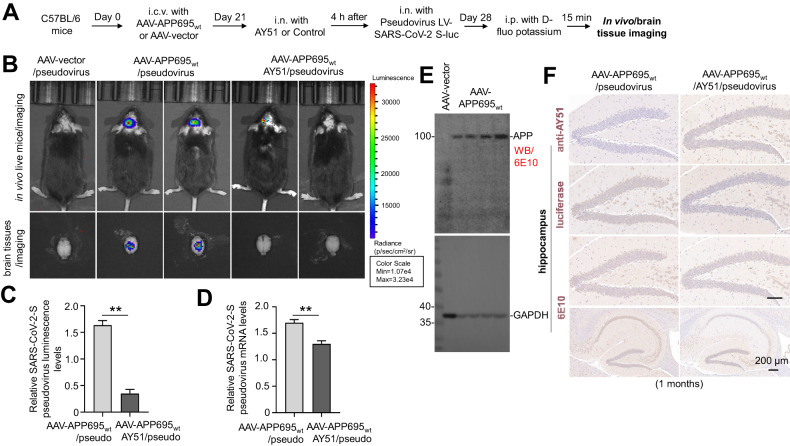
In vivo overexpression of human APP695wt promotes SARS-CoV-2 pseudovirus infection in C57BL/6J mice. **A** Schematic illustration of the experimental procedure. C57BL/6J mice at 1 month of age were intracerebroventricularly injected (interaural = 1.5 mm, Bregma = −2.5 mm, depth = 2 mm) with an adeno-associated viral vector (AAV)-encoding hAPP695wt (AAV-hAPP695wt) or AAV-empty vector (AAV-Vector) as control. Twenty-one days after injection, mice were treated intranasally (i.n.) with AY51 peptide (500 μg/kg, 5 μL) and then (4 h after treatment) infected with pseudovirus containing luciferase reporter gene via intranasal dropping (10^8^/mL, 2 μL). Seven days after pseudovirus exposure, AAV-hAPP695wt mice, AAV-hAPP695wt/AY51 mice, and AAV-vector control mice were intraperitoneally injected with d-luciferin (150 mg/kg body weight) and imaged with the IVIS Lumina III system within 15 min. After final imaging observation, all mice were sacrificed and their brains were separated immediately for immunohistochemistry (IHC) staining and qRT-PCR. **B** and **C** Dynamic changes of pseudovirus invasion in living mice were observed by using the IVIS Lumina III system. For further confirmation, brain tissues were carefully removed and imaged separately. Images were normalized to the same p/s range of 1.73e4–1.128e5 and displayed on the same color spectrum scale (right). Luminescence is observable in AAV-hAPP695wt mice; however, this can be reduced with prior treatment using AY51. Presented data represent the mean ± SD (*n* = 6) collected over three separate experiments, demonstrating significant differences (**P < 0.01). **D** Pseudovirus mRNA in the brain hemispheres homogenates of AAV-hAPP695wt mice and AAV-hAPP695wt/AY51 mice were quantified by qRT-PCR at 7 days post-infection. Pseudovirus can be detected in AAV-hAPP695wt mice, which can be diminished by pre-treatment with AY51. The data are shown as the means ± SD (*n* = 6) from three independent experiments (**P < 0.01). **E** RIPA-soluble fractions from brain hemispheres were analyzed for hAPP695wt expression in AAV-hAPP695 mice and AAV-vector control mice by WB using 6E10. **F** Pseudovirus and AY51 locations in 10-µm paraffin-embedded brain tissue section containing the hippocampus from AAV-hAPP695wt mice and AAV-hAPP695wt/AY51 mice were confirmed by immunhistochemical (IHC) staining using anti-AY51 antibody and anti-luciferease antibody (brown). hAPP695wt expression were also confirmed by 6E10 antibody (brown). Consistent with the data of qRT-PCR, clearly positive staining of luciferease can be observed, which can be significantly ameliorated by pre-treatment with AY51.

To further confirm the involvement of APP in the brain infection of pseudovirus in vivo, a mouse model overexpressing human wild-type APP695 was established by cerebral stereotactic injection of AAV-hAPP695wt in C57BL/6 mice. AAV-Vector was injected as a control. Twenty-one days after surgery, all AAV-APP695wt-injected mice were further administered N-terminal APP blocking peptide AY51 (intranasal, 500 μg/kg, 5 μL, detailed information of AY51 was described in Supplementary Fig. 4A–C), with saline as negative control. Four hours after peptides or saline administration, pseudovirus (luciferease-labeled) was exposed to all animals by intranasal drip. Seven days after pseudovirus exposure (on day 28), all animals were intraperitoneally injected with luciferin and subjected to luciferin imaging (Fig. 3A). Our data suggested that compared to AAV-vector control animals, clear brain infections of pseudovirus can be observed in AAV-APP695wt mice, as shown by the bright fluorescence signal (Fig. 3B, upper). Interestingly, in AY51-treated mice, brain infection of pseudovirus was significantly reduced (Fig. 3B, upper). Immediately after imaging, the brains were separated and further imaged by luciferin imaging. Consistent with the data from living animal imaging, the isolated brain tissues also showed clear fluorescence indicating the cerebral infection (Fig. 3B, lower). Moreover, the AY51 pretreatment significantly blocked pseudoviral infection in the brain as evidenced by the diminished fluorescence signal (Fig. 3B).

The brain tissues were further subjected to qRT-PCR assay to confirm the viral infection and the ameliorating effect of AY51 observed in luciferin imaging. Our qRT-PCR data revealed viral infection in brains and indicated that the relative luminescence and mRNA levels of SARS-CoV-2 pseudovirus in mouse brains were significantly decreased by AY51 treatment (*P* < 0.01, Fig. 3C and D). APP695wt overexpression induced by AAV-APP695wt was confirmed by WB (Fig. 3E) or IHC staining (Fig. 3F, lower panel). The AY51 distribution in the brain was also confirmed by IHC staining (Fig. 3F, upper). Interestingly, while the APP695wt levels were comparable between AAV-APP695wt mice and those AY51-treated AAV-APP695wt mice, AY51 treatment significantly reduced the luciferin-positive staining in the hippocampus of AAV-APP695wt mice (Fig. 3F, middle panel). All these findings indicated the involvement of APP in facilitating pseudoviral infection in mouse brains.

### Blockage of N-terminal APP inhibits SARS-CoV-2 pseudovirus infection in HEK293T/ACE2 and SARS-CoV-2 infection in Vero cells

**Fig. 4:**
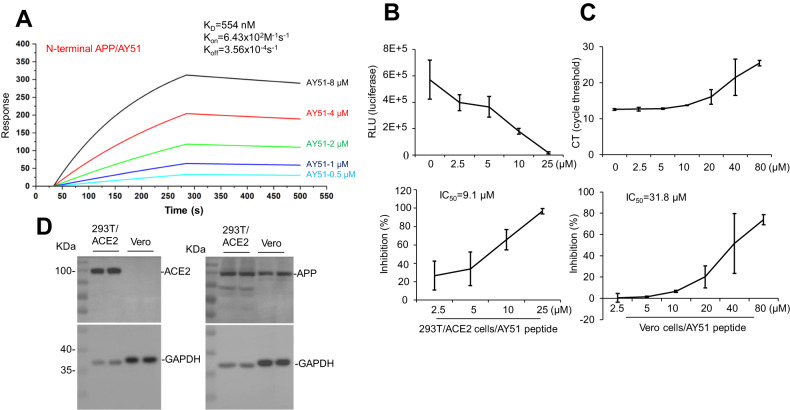
Blocking N-terminal APP inhibits SARS-CoV-2 pseudovirus infection in both HEK293T/hACE2 and Vero E6 cells. **A** N-terminal APP protein, captured on a COOH chip, can bind to AY51 with an affinity constant of 554 nM as determined by LSPR assay. **B** HEK293T/hACE2 cells at a density of 3 × 10^4^ cells/well were plated in 96-well plates and incubated with various concentrations of AY51 (0–25 µM) at 37 °C for 2 h, followed by adding the SARS-CoV-2 pseudovirus encoding luciferase for 72 h at 37 °C. Luciferase activity was measured by using a pseudovirus neutralization assay. The results are representative of three independent experiments, with each condition triplicated and presented as means ± SD of inhibition of the pseudovirus. **C** Vero cells at a density of 3 × 10^4^ cells/well were plated in 96-well plates for 24 h and infected with SARS-CoV-2 (at MOI of 0.05) with the presence of AY51 (2.5, 5, 10, 20, 40, and 80 μM). The virus yielded in the cell lysis was determined by qRT-PCR. Experiments were repeated twice, and the data are expressed as means ± SD. **D** The expression of ACE2 and APP protein in 293T/ACE2 and Vero cells was detected by WB.

To quantify the interaction of AY51 with N-terminal APP, an LSPR assay was employed via injecting various concentrations of AY51 (0.5–8 μM) to the LSPR sensor surface, on which APP18-612 fragment was pre-coated. As shown in Fig. 4A, data from the LSPR assay suggested the interactions of AY51 to APP18-612 in a concentration-dependent manner, with the KD value of 554 nM.

In HEK293T/ACE2 cells, AY51 peptide dose-dependently inhibited pseudovirus infection, evidenced by the decreased RLU signal, as assessed by luciferase activity assay (Fig. 4B, upper panel), with IC_50_ value at 9.1 μM (Fig. 4B, lower panel). At 25 μM concentration, AY51 completely blocked pseudovirus infection.

In African green monkey kidney Vero cells, qRT-PCR assay revealed that AY51 peptide inhibited SARS-CoV-2 infection in a dose-dependent manner (Fig. 4C, upper panel), with IC_50_ value at 31.8 μM (Fig. 4C, lower panel). Interestingly, while both HEK293T/ACE2 and Vero cells express APP protein (Fig. 4D, right), no ACE2 protein was detected in Vero cells by WB (Fig. 4D, left). This deficiency of ACE2 expression in Vero cells further confirmed the involvement of APP in SARS-CoV-2 infection.

### APP enhances SARS-CoV-2 pseudovirus infection in human brain organoids

**Fig. 5:**
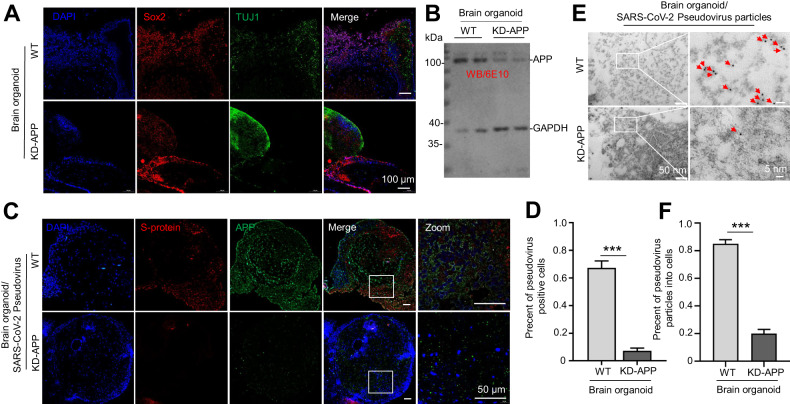
APP enhances SARS-CoV-2 pseudovirus infection in human brain organoids. **A** Human brain organoids were successfully constructed after inducing and culturing hiPSC cells for 40 days. The brain organoids with APP knockdown were constructed by using a lentivirus that continuously knocks down APP expression. Sox2 and TUJ1 antibodies were used to identify the successful expressions of regional cells and mature neurons in the ventricles of organoids. **B** WB assay was performed to confirm the expression of APP knockdown in the brain organoids infected by lentivirus. **C** Three days after SARS-CoV-2 pseudovirus infection in the organoid, the load of pseudovirus in the organoid was determined by immunofluorescent staining. **D** Quantitative analysis of pseudoviral load in (**C**). Statistical analyses for **C**, means ± SD; *n* = 6 for all groups; ***P < 0.001, Student’s *t*-test. **E** Immunoelectron microscopy (IEM) assay was conducted to verify the quantity of SARS-CoV-2 pseudovirus particles infiltrating the brain organoids infected by lentivirus. **F** Quantitative analysis was conducted on pseudoviral particles in group C. Statistical data are mean ± SD, with *n* = 6 per group. Significance was set at ***P < 0.001, via Student’s *t*-test.

According to the experimental protocol of the STEMCells kit, human brain organoids were successfully constructed after inducing and culturing human induced pluripotent stem cells (hiPSCs) for 40 days (Supplementary Fig. 2A). The brain organoids with low APP expression were constructed by using lentivirus that continuously knocks down APP expression (KD-APP) (Supplementary Fig. 2C). Sox2, Prox1, Foxg1, MAP2 and TUJ1 antibodies were used to identify the successful expressions of regional cells and mature neurons in the ventricles of organoids (Fig. 5A and Supplementary Fig. 2B). At the same time, compared with the wild-type brain organoids, the WB results showed that the expression of APP was significantly reduced in the brain organoids infected by KD-APP lentivirus (Fig. 5B). These results indicated the successful construction of organoid model and KD-APP organoid models.

Three days after SARS-CoV-2 pseudovirus infection in organoid, the load of pseudovirus in the organoid with low APP expression was significantly reduced (Fig. 5C, lower, and Fig. 5D, *P* < 0.001) evidenced by the reduced red fluorescent positive cells, as assessed by IF staining. At the same time, pseudovirus had regional differences in the infection of organoids (Fig. 5C, upper). Besides, immunoelectron microscopy showed that the number of pseudoviral particles entering the brain organoid models was also significantly reduced after APP knockdown (Fig. 5E, upper, and Fig. 5F, *P* < 0.001)

### Chronic SARS-CoV-2 pseudovirus infection enhances Aβ levels in vitro and in vivo

**Fig. 6:**
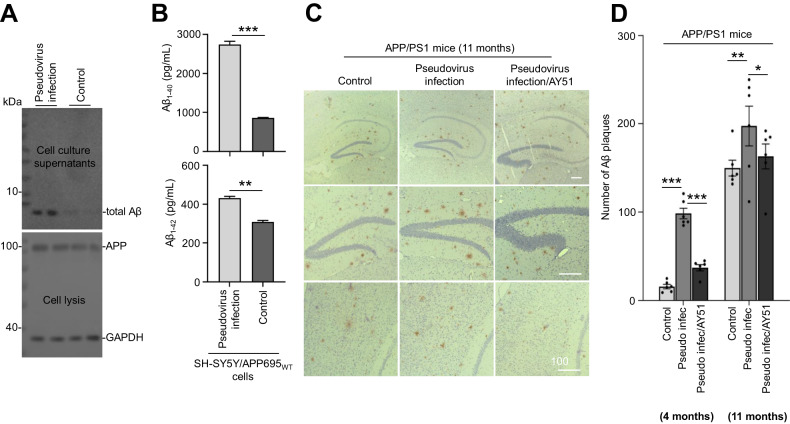
SARS-CoV-2 pseudovirus infection promotes Aβ production. **A** SH-SY5Y cells stably over-expressing APP695wt (SH-SY5Y/APP695wt) were plated in six-well chambers at a density of 1 × 10^5^ cells for 24 h and then incubated with pseudovirus at 10^10^/mL for 48 h. Total Aβ from cell lysis was detected by WB assay with 6E10. **B** The Aβ_1-40_ (upper) and Aβ_1-42_ (bottom) levels from the cell culture supernatants were quantified by Aβ enzyme-linked immunosorbent assay (ELISA). Results are representative of three independent experiments (mean ± SD. **P < 0.01; ***P < 0.001, one-way ANOVA with Bonferroni’s post hoc test (*n* = 6). **C** Representative anti-Aβ antibody (4G8) staining of cortical and hippocampal sections in APP^SWE^/PS1^ΔE9^ mice. Pseudovirus was intranasally dropped (i.n., 10^10^/mL, 2 μL) after AY51 treatment (i.n., 500 μg/kg body weight, 5 μL) in 2-month-old APP^SWE^/PS1^ΔE9^ mice (6 mice/group) and an empty lentiviral vector (LV-empty pseudovirus) as negative control. Aβ plaque was detected in cortical and hippocampal sections by immunohistochemistry (IHC) using an antibody (4G8) after 11 months (at 13 months of age). Scale bar 500 μm. **D** Quantitative analysis of the number of Aβ plaques in cortical (bottom) and hippocampal (upper) tissues in (**C**). Each slide was counted for Aβ plaques number via Image J software, and the number of the plaque was statistically quantified. Statistical analyses for **C**, means ± SD; *n* = 6 for all groups; *P < 0.05; **P < 0.01; ***P < 0.001, Student’s *t*-test.

Forty-eight hours after pseudovirus infection in SH-SY5Y/APP695wt cells, the cell culture medium supernatants were harvested for WB and ELISA. WB data indicated that total Aβ level was significantly elevated after pseudovirus infection (Fig. 6A). Consistently, ELISA data further suggested that pseudovirus infection induced dramatic increases of both Aβ_1-40_ (*P* < 0.001) and Aβ_1-42_ levels (*P* < 0.01, Fig. 6B).

In addition, pseudovirus was infected to 2-month-old APP/PS1 transgenic mouse model of AD. The number of amyloid plaques in the brain of E9 mice was observed after 4 and 11 months of infection. It was found that 4 or 11 months after infection, compared with the control group, the number of plaques in pseudovirus infected group increased significantly, which can be prevented by AY51 (Supplementary Fig. 3A for 4 months, Fig. 6C for 11 months, and Fig. 6D). Moreover, the IF staining revealed that 11 months after infection, pseudovirus infection-induced activation of astrocytes and microglia, as shown by the elevated astrocyte fibrillary acidic protein (GFAP) and microglial ionic calcium-binding junction protein molecule 1 (IBA-1) staining in the hippocampus of E9 mice. The results showed that pseudovirus infection could cause inflammation and significantly activate the expression of astrocytes and microglia in mouse brain (Supplementary Fig. 3B). Statistical analysis further indicated that pseudovirus infection could significantly expand the activated area of astrocytes and microglia in the hippocampus (Supplementary Fig. 3C, D).

## Materials and methods

### Cells lines and culture

HEK293T and SH-SY5Y cells were engineered to stably overexpress either human ACE2 (hACE2) or human APP695 via lentiviral transduction. This was achieved by co-transfecting HEK293T cells with a lentiviral transfer vector containing the coding sequence for hACE2 (GenBank: NM 021804.1) or hAPP695 (GenBank: NM 201414.3) named pLV-CMV-MCS-EF1-Puro, together with the packaging plasmids pMD2G (Catalog#12259; Addgene) and psPAX2 (Catalog#12260; Addgene) using Lipofectamine 2000 (Catalog#11668027; Invitrogen). Approximately 72 h after transfection, lentivirus-rich supernatants were harvested, pooled, and then used to transduce HEK293T or SH-SY5Y cells in a 1:1 mixture with culture medium and the aid of 8 μg/mL polybrene (Catalog#28728-55-4; GLPBIO). Following transduction, cells were selected for stable integration by culturing in a medium supplemented with either 1 μg/mL puromycin (Catalog#58-60-6; GLPBIO) (for HEK293T) or 2 μg/mL puromycin (for SH-SY5Y).

### Construction of SARS-CoV-2 pseudoviruses

For the construction of the PCDH-based pseudovirus, the pCDH-CMV-MCS-EF1 vector was digested using NotI and SalI enzymes, followed by gel purification of the product [42]. The Spike (N501Y) gene and EGFP gene were synthesized with overlapping ends via PCR, then cloned into the digested pCDH vector using Gibson assembly at 50 °C for 1 h (Catalog##E5510S; New England Biolabs) [43]. Subsequently, HEK293T cells were seeded in a 10-cm dish. After 24 h, the pCDH plasmid along with plasmids pMD2G (Catalog # 12259; Addgene) and psPAX2 (Catalog # 12260; Addgene) were packaged into HEK293T cells; Thermo Fisher Scientific), were transfected at a ratio of 4:3:1 using Lipofectamine 2000 (Catalog#11668027; Invitrogen). 72 h post-transfection, the lentivirus-containing supernatants were collected and centrifuged at 800×*g* for 5 min to remove cell debris. The supernatant was then subjected to ultracentrifugation (Himac CP100NX, Japan) at ~100,000×*g* for 2 h to concentrate the pseudovirus. Finally, the supernatant was removed, and the pelleted pseudovirus was then resuspended in an appropriate storage buffer and stored at −80 °C for use.

### Brain organoids culture

Undifferentiated hiPSCs were obtained from Beijing Cellapy Biotechnology with being verified pluripotent and contamination-free. hiPSCs were cultured in Matrigel (Catalog#356231, Corning) coated plates in feeder-free culture conditions with fresh PGM1 PSC culture medium (Catalog#CA1007500, Cellapy), and the cells were passaged at 80–90% confluence using a dissociation solution (Catalog#CA3001500, Cellapy). Culture medium kit for extended maturation of human cerebral organoids (Catalog#08570, STEMdiff). hiPSCs maintained in mTeSR1™ were dissociated into single-cell suspensions using Gentle Cell Dissociation Reagent (Catalog#100-0485, STEMdiff) and seeded at a density of 90,000 cells/well in a U-Bottom 96-well Ultra Low Attachment Plate (Catalog#CLS3474, Corning) containing Embryoid Body (EB) Formation Medium and Rho-Kinase Inhibitor (ROCKi). EBs were fed every 2 days with EB Formation Medium without ROCKi. After 5 days, EB was transferred to an Induction Medium in a 24-well Ultralow Attachment Plate (Catalog#CLS3476, Corning). EBs were cultured for an additional 2 days and were then embedded in liquid Matrigel (Catalog#CA3003100, Cellapy) followed by transfer to a non-tissue culture-treated six-well plate (5–8 organoids/well). Embedded organoids were maintained in Expansion Medium for 3 days. On Day 10, organoids were switched to Maturation Medium and cultured on an orbital shaker set at 65 rpm. Organoids were fed every 3–4 days with Maturation Medium. On Day 40, the forebrain cells or Cortical region cells of brain organoids (45-day-old) were immunostained with Sox2 (1:1000; Chemicon, AB5603) (red), Prox1 (1:1000; Chemicon, MAB5654) (red), Foxg1 (1:1000; Abcom, ab18259) (green), MAP2 (1:1000; Proteintech, 67015-1-lg) (red) and TUJ1 (1:1000; Covance, MMS-435P) (green) markers using antibodies. The brain organoids culture was schematically illustrated in Supplementary Fig. 2A–C. In order to obtain brain organoids with APP Knock-Down, Lentivirus (10 μL, 1 × 10^8^) containing APP-Homo-1551 shRNA (Genomeditech) was transfected into brain organoids for 72 h to knock down endogenous APP.

### Characterization of pseudovirus particles in brain organoids

Brain organoids were fixed with 4% polyformaldehyde, washed with PBS, and blocked with 1% BSA. Then, organoids were incubated with an anti-S antibody (1:2000; OriGene, TA890227), washed with PBS, and treated with a gold-labeled secondary antibody. Subsequent steps included another round of fixation, washing, and staining with uranyl acetate. The organoids underwent progressive dehydration and embedding in Epon 812 resin. Ultrathin sections were prepared, stained, and observed under a transmission electron microscope (JEM-1400FLASH, Japan). The infiltrating pseudovirus particles in the organoids were quantified and compared.

### Animals

All animal care and experimental procedures were performed according to the guidelines and were approved by the Animal Care and Use Committee of Guizhou Medical University. Mice (maximum of 6 mice per cage) were caged in a climate-controlled room (23 ± 0.5 °C) with 12 h of light and darkness with an ad libitum supply of food and water. Female C57BL/6 mice (12 weeks old) were procured from the Aniphe BioLab. Additionally, female APP/PS1 transgenic mice (8 weeks old), expressing human presenilin 1 (A246E) and Swedish mutant APP (APP_Swe_) were sourced from Jackson laboratory (Catalog#41848-JAX; B6C3-Tg (APP695)3DboTg (PSEN1)5Dbo/Mmjax). To ensure the welfare of the mice during certain procedures and prior to euthanasia, they were anesthetized using 4% isoflurane (Catalog#R510-22-10, Rayward Life Technology), bilateral thoracotomy and transcardial perfusion with physiological saline-containing heparin (10 U/mL) were then performed. Once these procedures were complete, the mouse brains were extracted. These specimens were then either reserved for biochemical analysis or were subjected to immunohistochemistry (IHC) staining. To ascertain the validity of the transgenic specimens, genotyping was meticulously carried out for each mouse, thus confirming the specific transgenic lineage.

### AAV injection and SARS-CoV-2 pseudovirus infection in C57BL/6 mice

AAVs, including AAV-APP695wt and AAV-vector, were produced and harvested as outlined in prior research [44, 45]. This engineered capsid enables efficient transduction of the CNS via the vasculature. Purified virus was concentrated, washed in PBS, sterile-filtered, and titred using quantitative PCR. pAAV-APP695wt and pAAV-vector constructs were packaged into AAV2/8. All procedures were performed with approval from the Institutional Animal Care and Use Committee and in compliance with the National Institutes of Health Guide for the Care and Use of Laboratory Animals, the Animal Welfare Act, and guidelines from the Guizhou Medical University. Female C57BL/6 mice at 12 weeks of age were intracranially injected with AAVs encoding AAV-APP695wt into the hippocampus (2.0 μL unilaterally) with AAV-vector as control. Injections were performed under standard aseptic surgery conditions. Mice were anaesthetized and maintained with 4% isoflurane and injections were made at the coordinates of interaural = 1.5 mm, Bregma = −2.5 mm, depth = 2 mm. A Hamilton needle was used to inject the AAV solution at a speed of 0.5 μL/min. Following injection, mice were allowed recuperation on a warming pad, receiving meloxicam (20 mg/kg) post-surgery for pain relief.

Refinements for intracranially introduced AAV-APP695wt dosages were derived from earlier studies [46]. AAV-APP695wt mice were then randomized into AAV-hAPP695wt/AY51 and AAV-hAPP695wt/saline control groups (6 mice per group). AAV-APP695wt/AY51 mice were injected intranasally with AY51 peptide (500 μg/kg, 5 μL), while AAV-APP695wt/saline mice were injected intranasally with saline. Four hours after peptides or saline treatment, all animals were intranasally injected with SARS-CoV-2 pseudovirus containing firefly luciferase gene (10 μL per mouse, 2 × 10^7^ copies/mL) [47]. On day 7 post the pseudovirus exposure (day 28), mice were intraperitoneally injected with luciferin (15 mg/mL, 200 μL per mouse). Immediately after luciferin or saline injection, animals were anesthetized by isoflurane and subjected to luciferin imaging using IVIS Lumina III in vivo imaging system (PerkinElmer, USA) to detect the invasion of pseudovirus in the brain. For further confirmation, brain tissues were carefully removed and imaged separately. Images were normalized to the same and displayed on the same color spectrum scale. The animal’s grouping and treatment were schematically illustrated in Fig. 3A.

### AY51 treatment and SARS-CoV-2 pseudovirus infection in APP/PS1 mice

At 8 weeks of age, female APP/PS1 double transgenic mice were treated with AY51 (administered intranasally at a dosage of 500 μg/kg body weight, 5 μL) and subsequently infected intranasally with SARS-Cov-2 pseudovirus (10^8^/mL, 2 μL) (6 mice/group). As a negative control, an empty lentiviral vector (LV-empty pseudovirus) was used. Aβ plaques were detected in cortical and hippocampal sections using the 4G8 antibody (1:1000; Biolegend, 800701) via immunohistochemistry (IHC) at 6 and 13 months of age. Furthermore, the expression of hAPP695wt in AAV-hAPP695 mice and their AAV-vector control counterparts was determined via Western Blot, utilizing the 6E10 antibody. In 10-µm paraffin-embedded brain tissue sections from AAV-hAPP695wt mice and AAV-hAPP695wt/AY51 mice—specifically those containing the hippocampus—the locations of the pseudovirus and AY51 were ascertained by IHC staining using the anti-AY51 antibody and anti-luciferase antibody (1:2000; orb1147993, Biorbyt), respectively. In addition, IHC staining with the 6E10 antibody also validated the expression of hAPP695wt.

### Localized surface plasmon resonance analysis

LSPR analysis was conducted using the Open SPR™ instrument (Nicoyalife, Canada). The COOH sensor chip was installed according to the manufacturer’s standard operating procedures. The buffer solution (PBS, pH 7.4) was run at the maximum flow rate of 150 μL/min until the signal baseline was achieved. The flow rate was then adjusted to 20 µL/min. A solution of 1-ethyl-3-(3-dimethylaminopropyl) carbodiimide combined with N-hydroxysuccinimide was loaded at a rate of 20 µL/min for 4 min to activate the COOH sensor chips. the interaction between anchored hACE2 or APP18-612 fragment and flowing SARS-COV-2 S protein by measuring changes in reflected light intensity. The concentrations of both proteins were optimized through prior tests for precise interaction dynamics [46, 48]. The concentrations of both proteins were optimized through prior tests for precise interaction dynamics. The injection port was rinsed with buffer solution and air-dried. A 200 µL blocking solution was introduced at a rate of 20 µL/min for 4 min. After rinsing and air-drying, the APP18-612 (expressed by KMD Bioscience) or hACE2 (Catalog#C419, Novoprotein) protein was loaded onto the sensor. A 5-min observation period ensured analytical stability. Subsequently, S protein (Catalog#SPN-C52H9, ACROBiosystems) or AY51 (synthesized by Qyaobio) was injected at various concentrations, from low to high. The kinetic parameters of the binding reactions, including Ka, Kd, and KD, were analyzed using the Trace Drawer software (Ridgeview Instruments AB, Sweden).

### Immunofluorescence staining

HEK293T/hACE2 or HEK293T/hAPP cells were plated in confocal chambers at a density of 1 × 10^5^/well for 24 h. After transfection with constructs encoding SARS-CoV-2 S protein for 48 h, the cells were fixed in 4% paraformaldehyde. Blocking was performed with 5% bovine serum albumin (BSA) in PBST (0.2% Triton × 100 in PBS) at 37 °C for 60 min. The chambers were then washed with PBS (pH 7.4). Immunofluorescence staining employed anti-hACE2 antibody (1:2000; Santa Cruz Tech, sc-390851), anti-hAPP antibody (1:3000; Biolegend, 803015), and anti-S protein antibody (1:2000; OriGene, TA890227). Alexa Fluor 488 (1:500; Invitrogen, A-11001) goat anti-mouse IgG detected hACE2 or hAPP (green), while Alexa Fluor 555 (1:500; Invitrogen, A-31572) donkey anti-rabbit IgG detected the S protein (red). Nuclei were counterstained with DAPI Fluoromount-G (Catalog#0100-01; Southern Biotech) and visualized using a confocal microscope (Olympus SpinSR10, Japan).

For immunofluorescence staining of astrocyte marker GFAP protein (1:2000; abcam, ab279289) and microglial marker IBA-1 protein (1:2000; abcam, ab178846) in hippocampal sections from APP/PS1 mice infected with pseudovirus or LV-empty pseudovirus, frozen sections between bregma −1.5 and −2.5 mm were prepared for each animal. Slides underwent antigen retrieval in a solution (Catalog # P0090, Beyotime) and were then washed with PBS (pH 7.4). Blocking was similar to the method for cell lines. Overnight incubation at 4 °C was done with diluted GFAP and IBA-1 antibodies in PBST. Alexa Fluor 488 (1:500; Invitrogen, A-11001) goat anti-mouse IgG detected GFAP (green), while Alexa Fluor 555 (1:500; Invitrogen, A-31572) donkey anti-rabbit IgG detected IBA-1 (red). Counterstaining with DAPI Fluoromount-G (Catalog#0100-01; Southern Biotech) revealed nuclear DNA (blue) for 10 min at room temperature. Slides were visualized as before. Image analysis was conducted with ImageJ software, which automatically analyzed the target signal area and calculated the percentage of the target signal.

### Co-immunoprecipitation

SH-SY5Y cells were co-transfected with SARS-CoV-2 S protein and APP constructs for 48 h. Cells were lysed using RIPA lysis buffer (Catalog#87787; Thermo Scientific) supplemented with a Halt protease inhibitor cocktail (Catalog#78425; Thermo Scientific). The lysates were clarified and precleared, after which protein concentrations were determined using the BCA protein assay kit (Catalog#23227; Thermo Scientific). To prepare the agarose bead-antibody complex, the S protein antibody or APP antibody was incubated with MAg25K protein A/G agarose beads (Catalog #FY60014; Enriching Biotech) for 1 h at 37 °C. Approximately 1/10 of the lysates were reserved for Western blot analysis. The remaining lysate was combined with the bead–antibody complex and incubated overnight at 4 °C. For Western blotting (WB), samples were separated by SDS–PAGE and transferred to PVDF membranes (Catalog#3010040001; Roche) using a semi-dry transfer method at 25 V for 30 min. Membranes were blocked with 5% skim milk in PBST for 1 h and then incubated overnight with an anti-APP antibody (1:3000; Biolegend, 803015) or anti-S protein antibody (1:2000; OriGene, TA890227) diluted in 5% BSA (Catalog#10711454001; Roche) at 4 °C. The membranes were then probed with HRP-conjugated secondary antibodies, either anti-mouse (Catalog#7076P2; Cell Signaling Technology) or anti-rabbit (Catalog#7074P2; Cell Signaling Technology), diluted in 5% milk. Protein bands were visualized and imaged using the GeneGnome XRQ system (USA).

### Western blotting

Cells were lysed using RIPA lysis buffer (Catalog#87787; Thermo Scientific) followed by sonication. Protein extracts were then collected from the supernatants after centrifugation. These extracts were separated by SDS–PAGE and transferred to membranes. After blocking with 5% BSA, the membranes were incubated with primary antibodies, followed by species-specific horseradish peroxidase-conjugated secondary antibodies. The membranes were subsequently washed with PBST three times. Protein bands were visualized using enhanced chemiluminescence reagents (Catalog#WBULS0100; Millipore) and captured using the GeneGnome XRQ system.

### Quantitative real-time PCR (qRT-PCR)

HEK293T/hACE2 cells transfected with constructs encoding hAPP, and HEK293T/hAPP cells transfected with constructs encoding hACE2, as well as SH-SY5Y, SH-SY5Y/APP695wt, and SH-SY5Y/KD-APP, were infected with the GFP-labeled pseudovirus at a concentration of 10^10^/mL and incubated for 72 h at 37 °C. Meanwhile, AAV/Vector mice, AAV-APP695wt mice, and AAV-APP695wt/AY51 mice were infected with the GFP-labeled pseudovirus at 10^10^/mL for 7 days. Vero E6 cells were plated at a density of 3 × 10^4^ cells/well in 96-well plates and were incubated for 24 h. These cells were then infected with SARS-CoV-2 at an MOI of 0.05 in the presence of various concentrations of AY51 (2.5, 5, 10, 20, 40, and 80 μM). The amount of pseudovirus present in the cell lysate was quantified using qRT-PCR.

Total RNA was extracted from either the cell lysates or homogenized brain tissues using TRIzol reagent (Catalog#15596026, Invitrogen) as per the manufacturer’s instructions. The extracted RNA was then reverse-transcribed into cDNA using the Prime Script II 1st Strand cDNA Synthesis Kit (Catalog#6210A, TaKaRa) on the T100 PCR system (Bio-Rad, USA). The resulting cDNA was amplified using specific primers (listed in Table S1) and the TB Green^®^ Fast qPCR Mix (Catalog#RR430A; TaKaRa). The relative expression levels of the pseudovirus were measured using the CFX Connect Real-Time System (Bio-Rad, USA).

### Immumohistochemical staining (IHC)

Number of plaques in APP/PS1 mice (AAV-vector/pseudovirus, AAV-APP695wt/pseudovirus, and AAV-APP695wt/AY51/pseudovirus) was quantified after 4 and 11 months of pseudovirus infection. Mice were perfused with 4% paraformaldehyde in 0.1 M sodium cacodylate (pH 7.4) for 15 min at room temperature. Subsequently, the brains were immersion-fixed for 24 h at the same temperature. Immunocytochemistry was performed following a protocol described elsewhere. Briefly, samples were rinsed three times for 5 min each and once for 1 h in cold phosphate-buffered saline (PBS, pH 7.4), and then sectioned to 100 µm using a vibratome (Leica, Germany). Sections were immersed in normal goat serum (Catalog#abs933; absin) at a 1:10 dilution in PBS containing 0.5% BSA, 0.1% Triton-X 100, and 0.1% sodium azide (PBTA). This was kept at 4 °C on a rotator with overnight agitation. Sections were then treated with primary antibodies against the S protein (1:1000 dilution; Invitrogen, PA5-116916) in PBTA. Following this, sections underwent rinsing in PBTA five times for 5 min each and once for 1 h. They were then exposed to the corresponding secondary antibodies. Finally, sections were rinsed and mounted on a glass slide using DAPI Fluoromount-G (Catalog#0100-01; Southern Biotech).

### Enzyme-linked immunosorbent assay (ELISA)

To quantitatively analyze the production of Aβ40 and Aβ42 in SH-SY5Y/APP695wt cells post-infection with SARS-CoV-S pseudovirus, the levels of Aβ40 and Aβ42 in the supernatant from cultured SH-SY5Y/APP695wt cells were determined. The Aβ40 (Catalog#KHB3481, Invitrogen) and Aβ42 (Catalog#KHB3442, Invitrogen) ELISA kits were used following the manufacturer’s instructions.

### Statistical analysis

All statistical analyses and graphical illustrations were executed using GraphPad Prism 9. The data obtained from the experiments were presented as mean ± SEM, derived from at least three independent experiments. For comparisons between the two groups, the two-tailed Student’s *t*-test was employed. For more than two groups, a one-way ANOVA, followed by Bonferroni’s post hoc tests, was used. Specifically, qRT-PCR and ELISA datasets were analyzed using a two-way ANOVA with Bonferroni’s post hoc tests. Adjusted *p*-values from multiple comparisons were calculated and are noted in the figure legends. A *p*-value of less than 0.05 was considered statistically significant. For clarity, the following notations were used: *p < 0.05, **p < 0.01, ***p < 0.001.

## Discussion

Given the increasing reports of neurological symptoms associated with COVID-19, understanding viral tropism is of significant interest for the development of better treatments and for the prevention of long-term adverse impacts of infection. Our findings indicate that SARS-CoV-2 infects cells through APP-mediated mechanisms, leading to exacerbated Aβ pathology and neuroinflammation, as evidenced by cell culture, cerebral organoids, and animal models. This finding is consistent with the high levels of expression of APP in the CNS, as well as recent data from neural organoids showing minimal neuronal susceptibility to SARS-CoV2 and rather efficient CNS infection leading to transcriptional deregulation and cell death [30].

Despite the amyloid pathology, tau pathology has also been correlated with SARS-CoV-2 infection. Recently, using SH-SY5Y neuroblastoma cells and K18-hACE C57BL/6J mice, Di Primio et al. found that SARS-CoV-2 infection promotes tau phosphorylation and increases tau aggregation [49]. In addition, hyperphosphorylated tau was further observed in the cortex of the coronavirus mouse model of COVID-19 even 12 months post-infection [50]. This long-term impact might be mediated by the infection-induced inflammation and tau mislocation [31, 51]. Clinically, patients with COVID-19 without substantial neurological symptoms showed significantly higher plasma concentrations of GFAP, a marker of astrocytic activation, and of NfL and total tau, markers of axonal damage and neuronal degeneration, compared with controls [52]. Consistently, a large cohort study found that ~34% of patients received a psychiatric or neurological diagnosis within 6 months of SARS-CoV-2 infection [53]. In addition, a postmortem study revealed activation of neuropathological pathways causing tau hyperphosphorylation by COVID-19 infection [22].

A likely interpretation of the vulnerability of the brain to SARS-CoV-2 infection is mainly due to an indirect, secondary consequence of viral infection, such as the disrupted blood–brain barrier [54–56], rather than neurons themselves. Previous studies have demonstrated a leakage of blood proteins into CSF in more than 40% of patients tested [57]. Another possible route for the cerebral invasion of SARS-CoV-2 is through the nose [58, 59]. However, the exact mechanisms underlying the neuronal invasion of SARS-CoV-2 are rarely investigated.

In our study, we discover APP as a new receptor for SARS-CoV-2 infection. It has been reported that SH-SY5Y cells can be infected with SARS-CoV-2, which is also confirmed in our study. However, the introduction of APP alters the virus infectivity toward SH-SY5Y cells. APP as a new receptor is also approved by loss and gain function approaches and neutralization activity of APP extracellular domain. Another meaningful finding in our work is that iPSCs-induced cerebral organoids can

be infected with SARS-CoV-2 pseudovirus, in which knockdown APP expression decreased the virus infection. The endocytosis of APP is reported to be elevated in inflammatory nerve cells. In the meanwhile, the expression level of APP is higher than that of ACE2 in SH-SY5Y cells and no expression of ACE2 is detected in Vero E6 cells. Interestingly, the immunofluorescent assay shows no co-localization of APP and ACE2 in brain tissues from COVID-19 patients, as well as no interaction in detected cells. These findings suggest that APP and ACE2 may be two complementary receptors in mediating virus infection.

In our present study, activated expression of glial cells was observed following the hyper-production of Aβ. Neuroinflammation is another important CNS feature of SARS-CoV-2 infection. It is interesting to note that among the neurological disturbances seen in this and previous CoV outbreaks, chronic fatigue, and nonrestorative sleep disturbances have been noted. Indeed, multiple viruses, such as HCV, HIV, and EBV, have been linked to chronic fatigue syndrome (CFS), also known as myalgic encephalomyelitis (ME) [60–62]. While the exact cause of CFS/ME is still obscure, abnormal levels of a number of cytokines, such as IL-10 have been observed in CSF from patients with CFS/ME, suggesting an imbalance in neuroimmune modulation. In HCV in particular, the most common extrahepatic symptom in patients is chronic fatigue, and key mediators of HCV assembly are apolipoproteins with regulation of lipoprotein metabolism being important for the HCV replication cycle.

In conclusion, we have identified APP as a receptor protein for nerve cells and revealed a previously unknown mechanistic insight into COVID-19-related neuropathological sequelae. A systematical examination of multiple Omicron sub-variants on potential brain dysfunction would be warranted in future studies. The Spike protein could function as an immune switch to Aβ production and contribute to neurological changes in COVID-19 patients.

## Availability of data and materials

The data and materials that support the findings of this study are available from the corresponding author upon reasonable request.

### Supplementary information

Supplementary Figures legend

suppl Fig. 1

suppl Fig. 2

suppl Fig. 3

suppl Fig. 4

supplementary table 1
