# Supplementary material for: Amyloid precursor protein facilitates SARS-CoV-2 virus entry into cells and enhances amyloid-β-associated pathology in APP/PS1 mouse model of Alzheimer’s disease
Source: Transl Psychiatry. 2023 Dec 16;13:396. doi: 10.1038/s41398-023-02692-z (PMC10725492; doi:10.1038/s41398-023-02692-z)
Supplement: Supplementary file 1 — Supplementary Figures legend [file 41398_2023_2692_MOESM1_ESM.docx]

**Supplementary information**

**AY51 peptide design and synthesis**

We previously designed a series of peptides named as AYxx with potential binding activities with ACE2, APP or S-protein of SARS-CoV-2. Among these peptides, AY51 (KKKKKKLKVRLASHLRK(Pal)ELRKRLLRDA) is a peptide derived from ApoE protein (ApoE133-152) with two modifications on the N-terminal with 6 lysines (K) and the residue of K17 with palmitoylation (Pal). In our previous study, the parent peptide 6KApoEp (KKKKKKLKVRLASHLRKELRKRLLRDA) shows a binding ability to the N-terminal APP and blocks the interactions between N-terminal APP and its ligands (Sawmiller D et al. Biol Psychiatry. 2019; 86(3): 208-220). AY51 is designed via palmitoylation on K17 of 6KApoEp to enhance its bioavailability without impacts on its biofunction as N-terminal APP antagonist. AY51 was synthesized commercially by Novoprotein Scientific Inc. (Suzhou, China).

**AY51-antibody production and specificity confirmation**

AY51 polyclonal antibody was produced commercially by Absin Bioscience Inc. (Shanghai, China). The antigen used for antibody production is LKVRLASHLRKLRKRLLRDA. The specificity of antibody was further confirmed by WB and IHC staining. As expected, the antibody canrecognize AY51 both in vitro in cell cultures and in vivo in mouse brain (**Suppl. Fig. 4A-4C**).

**Suppl. Figure 1 SARS-CoV-2 S protein highly binds to N-terminal APP**

**B-C** BSA and tau were utilized as blank controls. **A** Concurrently, an irrelevant immobilized protein, ApoE, served as the negative control. Soluble proteins were introduced into cells, followed by co-localization observation via immunofluorescence. Notably, significant co-localization with Spike (S) protein was observed both on the surface and inside cells expressing ACE2 or APP. **D** In contrast, Caveolin-1 (CAV1), serving as a negative control, showed no co-localization with the S protein.

**Suppl. Figure 2 Culture and identification of human brain organoids**

By inducing differentiation of human induced pluripotent stem cells (iPSCs) using exogenous morphogens and neurotrophic factors in the developmental order of the cerebral cortex, a stable 3D in vitro culture system for creating human brain organoids was successfully created. **A** iPSCs were dissociated into single-cell suspensions using gentle cell dissociation reagent (GCDR), counted, centrifuged, resuspended in EB formation media with 10 mM Rho kinase inhibitor (ROCK), and plated on 96-well round-bottom ultra-low attachment culture plates. The plates were then put in an incubator with 5% CO2 and 37°C to continue culture. On days 2 and 4, each well received the proper amount of EB formation media. On day 5, embryoblasts were viewed under an inverted microscope before being moved to 24-well ultra-low attachment culture plates and cultivated in induction media. On day seven, the EBs were harvested, embedded in Matrigel, and transferred to 6-well ultra-low attachment culture plates (12 to 16 EBs per well) containing expansion media. On day10, the expansion medium was replaced with maturation medium, and the brain organoids were cultivated further in a spinning bioreactor with centrifugal force, with medium changes every 3-4 days. Scale bar, 1 mm. **B** On day 40, the brain organoids were frozen and sectioned for further immunofluorescence staining analysis of the expression of target proteins, including cortical granule cell precursor cells and pyramidal cells (Prox1), The early cortical neurons (Foxg1), cortical plate neurons (MAP2 and TUJ1). Scale bar, 5 μm. **C** The brain organoids (40 days) with low APP expression were constructed by using SARS-CoV-2 pseudovirus (1x 10^7^ IU/mL) that continuously knocks down APP expression (KD-APP). 3 days after SARS-CoV-2 pseudovirus infection in organoid, the load of pseudovirus in the organoid with low APP expression was significantly reduced.

Scale bar, 1 mm.

**Suppl. Figure 3 SARS-CoV-2 pseudovirus infection promotes Aβ production and microglial /astrocyte activation**

**A** Representative anti-Aβ antibody (4G8) staining of paraffin-embedded brain tissue sections in APP^SWE^/PS1^ΔE9^ mice. Empty lentiviral vector pseudovirus (LV-empty pseudovirus) as control, pseudovirus after AY51 treatment (500μg/kg, 5 μL) and psedovirus were intranasally dropped (10^8^/mL, 2μL) in 2-month-old APP/PS1ΔE9 mice. **B** Aβ plagues were detected by antibody (4G8) in cortical and hippocampal sections by immunohistochemistry (IHC) using antibody (4G8) at 6 months of age. Scale bar, 100 μm. Representative immunofluorescence of astrocytpe marker GFAP protein and microglial marker IBA-1 protein in hippocampal sections of pseudovirus or LV-empty pseudovirus in APP/PS1ΔE9 mice at 13 months of age (11 months after pseudovirus infection at 2 months of age). Alexa Fluor 488 goat anti-mouse immunoglobulin G (IgG) was used to detect GFAP (green), while Alexa Fluor 555 donkey anti-rabbit IgG was used to detect IBA-1(red). DAPI (40,6-diamidino-2-phenylindole) counterstain showed nuclear DNA (blue) and visualized by confocal microscopy. Scale bar, 100 μm. **C-D** Quantification of percentage of GFAP (upper) and IBA1 (bottom) area in **B**. Statistical analyses for **B**, means ± SD; n=6 (pseudovirus group) or n=6 (control group); **P* < 0.05, ****P* < 0.001, Student’s *t*-test.

**Suppl. Figure 4 AY51-antibody confirmation**

**A** Stability of AY51 peptide in HEK293T cell culture as assessed by WB. HEK293 cells were cultured with the presence of AY51 peptide (10 uM) for 0-360 min. At different time points, cell lysis was collected and subjected to WB (anti-AY51 antibody, 1:6000). **B** AY51 antibody confirmation by WB in N2a supernatant. N2a cells were co-incubated with various concentrations of AY51 peptides (10 uM and 30 uM) for 24 h. Supernatant was collected and subjected to WB analysis (anti-AY51 antibody, 1:6000). Lane 1: N2a cell supernatant containing AY51 (10 uM); Line 2: N2a cell supernatant (control); Line 3: N2a cell supernatant containing AY51 (30 uM). **C** The distribution of AY51 peptide in brain tissue and the specificity of the AY51 antibody as assessed by immunohistochemistry staining 14 days after intranasal AY51 treatment (5uL, 2ug/uL) in C57BL/6 mice. Biotin antibody was used as control.
