# Supplementary figures and images for: Amyloid precursor protein facilitates SARS-CoV-2 virus entry into cells and enhances amyloid-β-associated pathology in APP/PS1 mouse model of Alzheimer’s disease
Source: Transl Psychiatry. 2023 Dec 16;13:396. doi: 10.1038/s41398-023-02692-z (PMC10725492; doi:10.1038/s41398-023-02692-z)

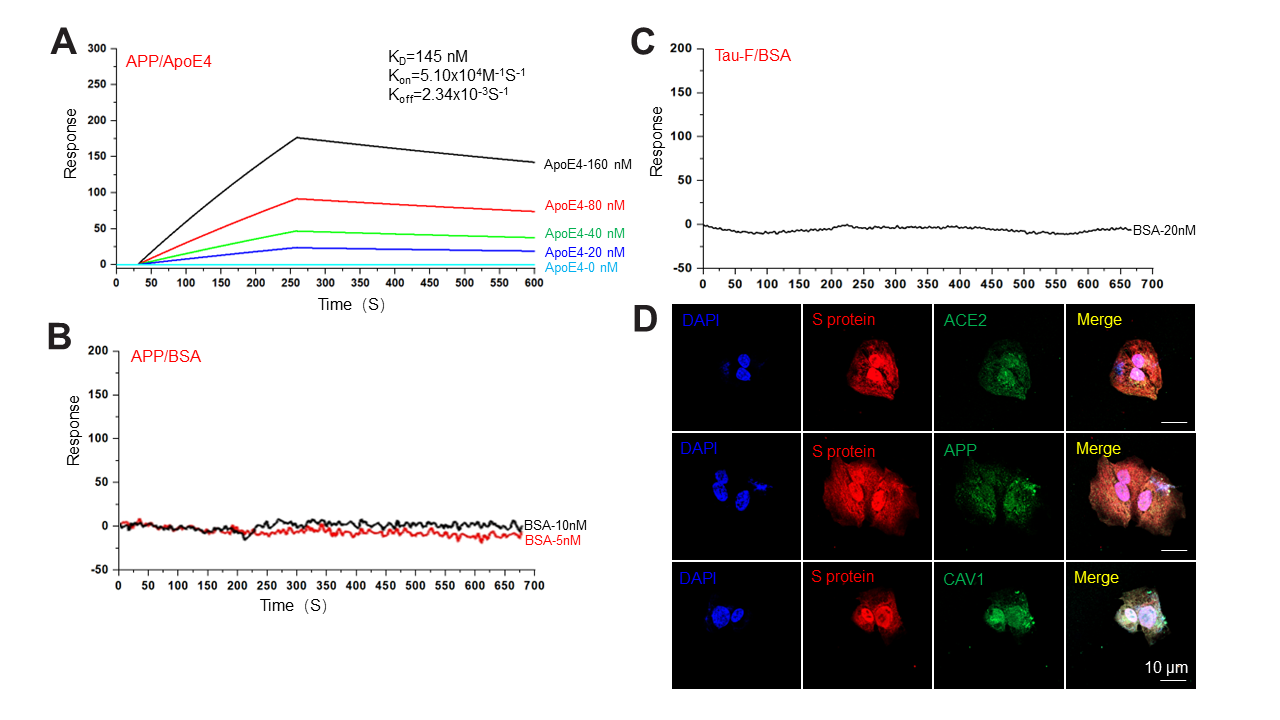

Supplement: Supplementary file 2 — suppl Fig. 1 [file 41398_2023_2692_MOESM2_ESM.tif]

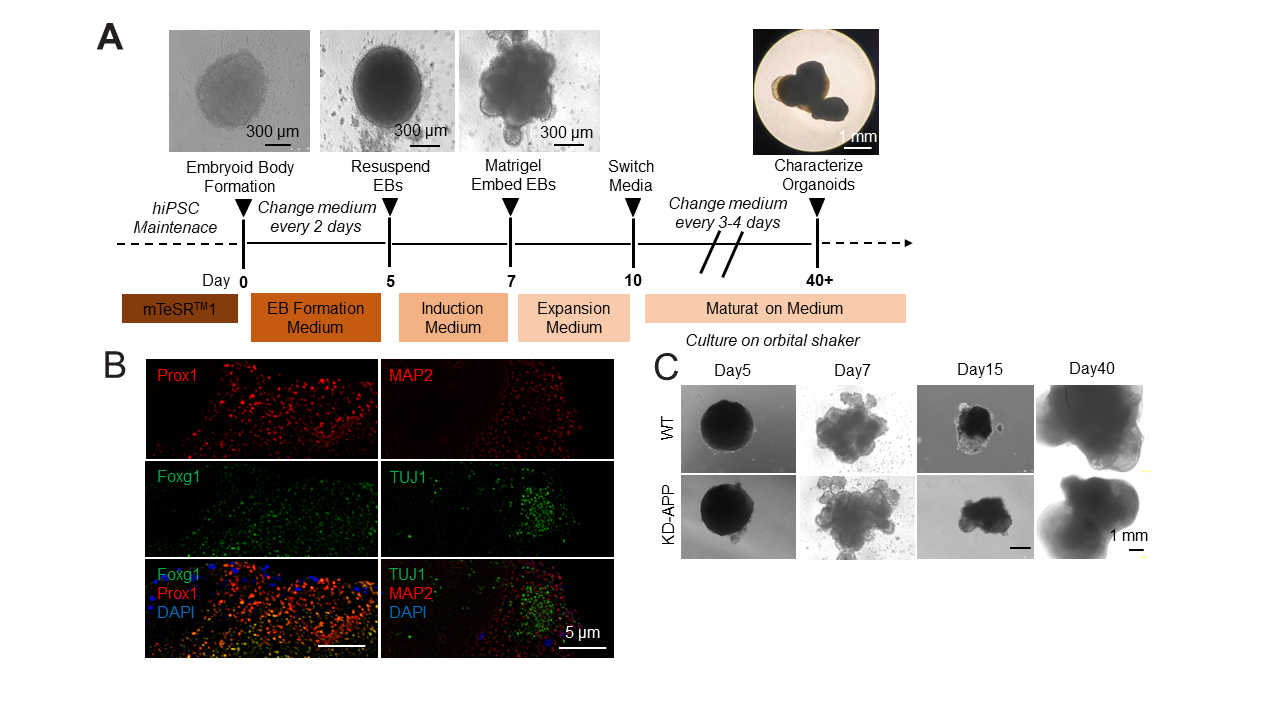

Supplement: Supplementary file 3 — suppl Fig. 2 [file 41398_2023_2692_MOESM3_ESM.tif]

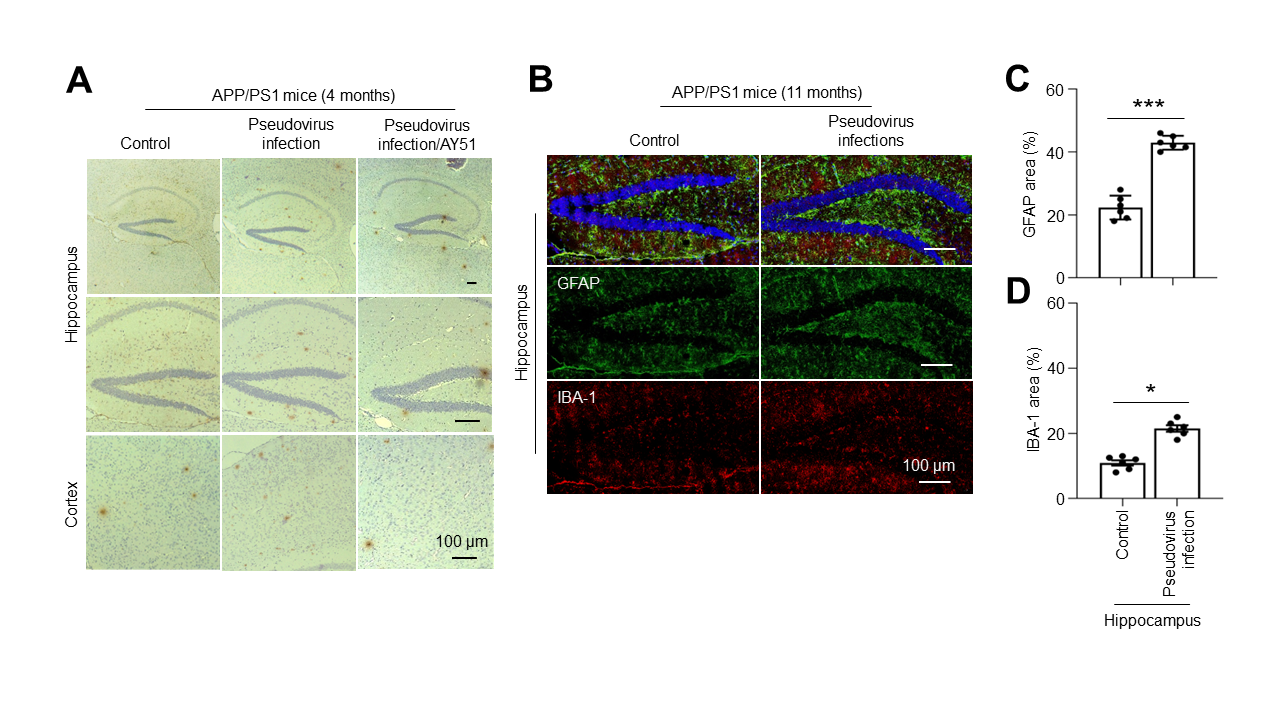

Supplement: Supplementary file 4 — suppl Fig. 3 [file 41398_2023_2692_MOESM4_ESM.tif]

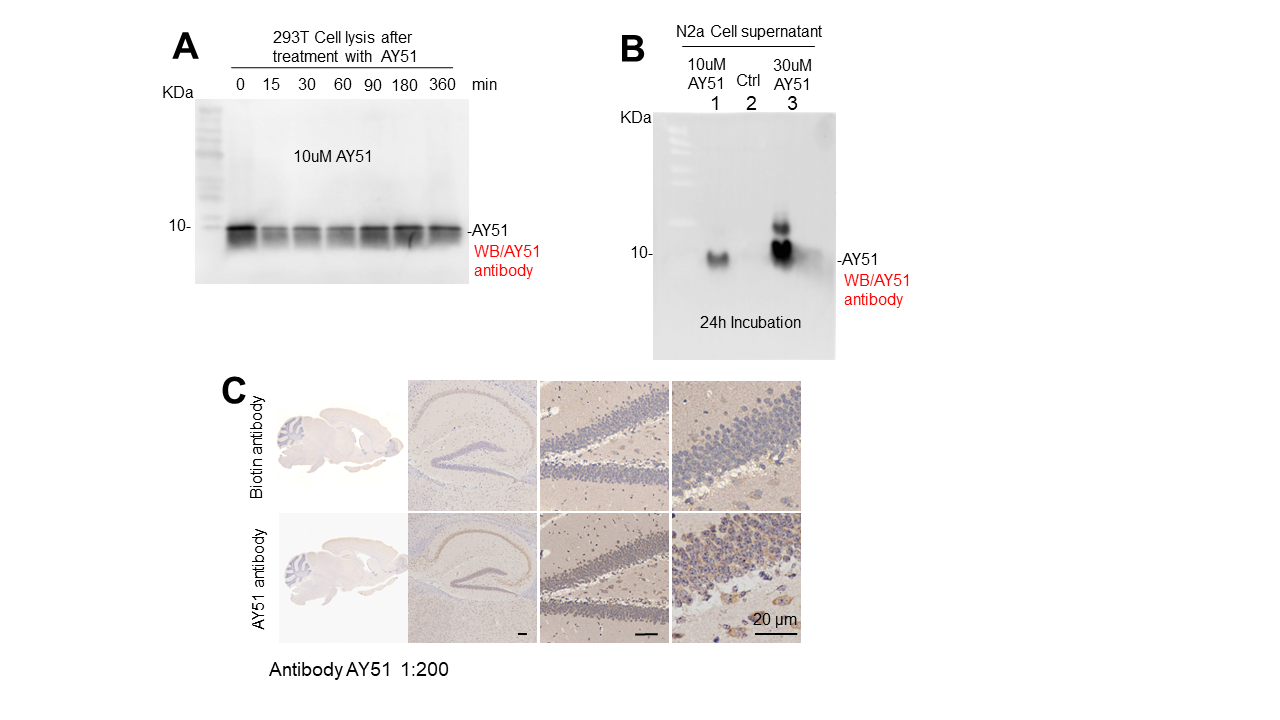

Supplement: Supplementary file 5 — suppl Fig. 4 [file 41398_2023_2692_MOESM5_ESM.tif]
