# Supplementary material for: Amyloid precursor protein facilitates SARS-CoV-2 virus entry into cells and enhances amyloid-β-associated pathology in APP/PS1 mouse model of Alzheimer’s disease
Source: Transl Psychiatry. 2023 Dec 16;13:396. doi: 10.1038/s41398-023-02692-z (PMC10725492; doi:10.1038/s41398-023-02692-z)
Supplement: Supplementary file 6 — supplementary table 1 [file 41398_2023_2692_MOESM6_ESM.docx]

Table S1 Sequences of qRT-PCR primers

| **Name** | **Sequences of primer (5’ to 3’)** |
| --- | --- |
| APP-F | CTCAGATCCGGTCCCAGGTT |
| APP-R | TCCTGAATCTCCTCGGCCAC |
| ACE2-F | CATTGGAGCAAGTGTTGGATCTT |
| ACE2-R | GAGCTAATGCATGCCATTCTCA |
| Psedovirus-F | ATTGCCACGGCGGAACTC |
| Psedovirus-R | AGCAGCCAAGGAAAGGACGA |
| GAPDH-F | AGGTCGGTGTGAACGGATTTG |
| GAPDH-R | TGTAGACCATGTAGTTGAGGTCA |
| N protein-F | TGGAATTCTGTCGTTGAGGGT |
| N protein-R | TCCTACGGGAGGCAGCAGT |
